# Effects of mobile phone-related distraction on driving performance at roundabouts: Eye movements tracking perspective

**DOI:** 10.1016/j.heliyon.2024.e29456

**Published:** 2024-04-10

**Authors:** Wafa Boulagouas, Ortega Carlos Alberto Catalina, Miguel Angel Mariscal, Sixto Herrera, Susana García-Herrero

**Affiliations:** a Institute of Health and Safety, University of Batna 2, Batna, Algeria; b Escuela Politécnica Superior, Universidad de Burgos, Burgos, Spain; c Department of Applied Mathematics and Computer Sciences, Universidad de Cantabria, Santander, Spain

**Keywords:** Health and safety promotion, Mobile phone, Driving performance, Roundabout, Eye movement, Driving simulator

## Abstract

Modern road infrastructures are complex networks featuring various elements such as roads, bridges, intersections, and roundabouts, with advanced control systems. Roundabouts have gained prominence as a safer alternative to traditional intersections promoting smoother traffic flow and fewer collisions by guiding traffic in one direction, encouraging reduced speed, and minimizing conflict points.

This study investigated driver behavior within roundabouts, focusing on gaze behavior, particularly the left-side mirror and window, under mobile phone distraction conditions. In addition, the effects of roundabout specifications (i.e., number of lanes and size of the central island) and the drivers’ characteristics (i.e., driving experience) were examined.

In total, 43 participants, aged 19–56 years including 30 males and 13 females, held a valid driving license, drove through a virtual simulated urban road containing four roundabouts, implemented in a static driving simulator, under baseline condition (no distraction) as well as mobile-induced distraction. Driving simulator data were collected and drivers’ gaze direction and fixation on nine areas of interest were captured with an eye tracker.

**Results:**

showed that experienced drivers exhibit a more fixation on the left-side mirror and window and were less distracted. Moreover, the road environment, i.e., the number of cars and the roundabout size, significantly influenced the drivers’ attention. As regards the driving performance, the number of infractions increased when the drivers diverted focus from the left side of the car. The outcomes of the present study might help to improve traffic safety at roundabouts.

## 1 Introduction

Traffic crashes have far-reaching consequences, including health problems, increased rates of impairments and disabilities [1,2] Along with significant losses in productivity, reduced mobility, and adverse effects on economic development and growth prospects [3,4].

Inattentive driving and multitasking are major contributors to road crashes and fatalities with distraction being a common factor in a significant portion of traffic crashes [5,6]. Moreover, previous studies analyzing road crashes associated with distracted driving and multitasking asserted that drawing attention away from driving leads to increased steering variability, lateral position deviation, aggressive braking, and delayed responses [[7], [8], [9]].

Recent road safety research has focused on the analysis of the effects of mobile phone use at the wheel, emphasizing factors that may adversely affect driver performance. In this regard, behavioral research reported that both genders engage in mobile phone use while driving at similar frequencies [10,11] although slight differences were noted [12]. Additionally, young adults were found to experience the greatest distraction and were involved in a significant number of crash-related fatalities [13,14].

In the same context, certain roadway configurations, such as intersections and roundabouts, while offering benefits such as increased capacity, safer traffic flow, enhanced mobility and reduced conflict points and crash rates [[15], [16], [17], [18]], they present unique complexities for drivers and further under mobile phone distraction. Engaging in a secondary task (i.e., use of mobile phone) results in visual, cognitive and yet physical impairments that affect appropriate decisions and potentially influence the drivers ability to safely travel through roundabouts [19,20]. Moreover, Haque et al. [20] emphasized the deceleration at faster rates before gap acceptance behavior of distracted drivers, which can compromise safety. Furthermore, Ortega et al. [21] and Al Aufi et al. [22] investigated the driving performance under mobile phone induced distraction at roundabouts, and reported significant differences in the driving performance and traffic violations between distracted and non-distracted drivers. Observational studies further indicated that about one-third of crashes at roundabouts are due to vehicle-control impairments, misjudgments, and failure to give way [[23], [24], [25]].

Navigating roundabouts requires simultaneous responses to various stimuli on the road, including other road users driving behaviors, and complex environments and layouts, such as central island, curb, lanes [[26], [27], [28]]. Moreover, when entering a roundabout, drivers must check for oncoming vehicles, vehicles ahead, cyclists, and pedestrians [29] and contend with continuous changes in visual perspective, which can lead to dangerous situations or traffic crashes, especially for cyclists and motorcyclists who are often overlooked by the drivers when entering or exiting roundabouts [30,31].

Even though there has been a considerable number of studies conducted examining different aspects of driving through roundabouts and assessing the safety performance of the drivers (A summary of previous research is provided as a supplementary file), to the best of the author's knowledge, direction and fixation on left-side mirror and window under mobile phone distraction and the subsequent effect on the drivers' performance received limited attention, although the gaze at these two areas is crucial to safe travel through a roundabout. Thus, this study builds on Azimian et al. [32] work and sets out to extend its research context to examine fixation on the left-side mirror and window and compare the drivers' performance under baseline and mobile phone-induced distraction.

Moreover, it is evident that the geometric design and characteristics of roundabouts, i.e., the number of lanes, and the size of the central island, have a significant effect on the driving behavior of the drivers. Thus, this study examines the potential influence of different roundabout configurations on the drivers’ gaze behavior and driving performance.

Finally, as drivers’ characteristics were found to be associated with risky behaviors when distracted by mobile phone use, this research compares the gaze behavior of experienced and novice drivers and their driving performance when steering through roundabouts, under baseline and mobile phone-induced distraction.

## 2 Material and methods

This section details the general set-up, design, and overview of the study.

### 2.1 Apparatus

#### 2.1.1 Driving simulator

**Fig. 1:**
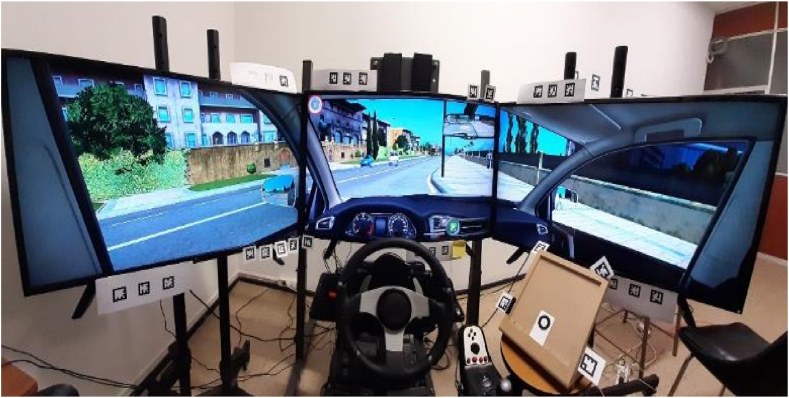
Driving simulator.

To conduct this study, driving performance data was gathered using an on-campus adapted DriveSim driving simulator (Fig. 1). The simulator comprises three connected screens, each sized 39″ with a resolution of 1920 × 1080 resolution providing a proper field of view of the simulated traffic, road environment, and weather conditions. Driving scenes are recorded with an update rate of 40–60 Hz given the hardware's graphics overload capability in the virtual reality environment.

For dynamic simulation, the apparatus is equipped with an accelerator, brake and clutch pedals, gear system, turn signals, a steering wheel, etc., and uses sophisticated artificial intelligence applications to mimic a variety of driving situations under different conditions.

**Fig. 2:**
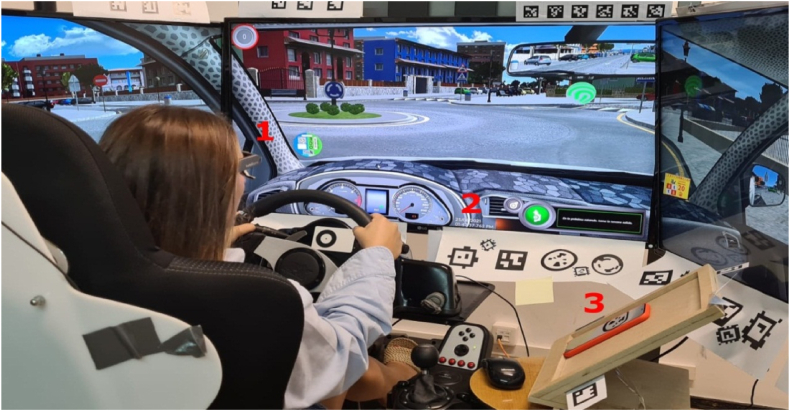
Additional devices used (Numbers identify locations of the devices: **(1)** eye tracker, **(2)** timestamp, and **(3)** mobile phone).

For this study, further devices have been included (Fig. 2): (1) an eye tracker to follow the driver's gaze, (2) a timestamp, and (3) a mobile phone placed to the right of the steering wheel.

#### 2.1.2 Timestamp

**Fig. 3:**
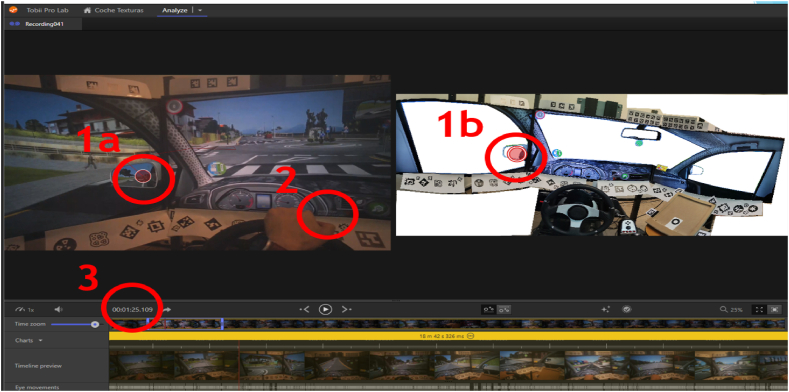
**(1a)** eye fixation in a video recorded by the eye tracker; **(1b)** position around the vehicle's cockpit as detected by the Tobii Pro Lab software, in this case, the left mirror; **(2)** timestamp within the simulator that enables the synchronization with the eye tracker timestamp; **(3)** eye tracker timestamp that allows computing the difference between both systems.

To synchronize the simulator data and the eye tracker, a separate timer is added to the simulator. This timer displays the minutes, seconds, and milliseconds of each frame rendered by the simulator to be used in the database. Thus, the time synchronization of the entire system used the records from the 43 experiments in which the timestamp of the simulator was distinctly visible in the records (Fig. 3).

**Fig. 4:**
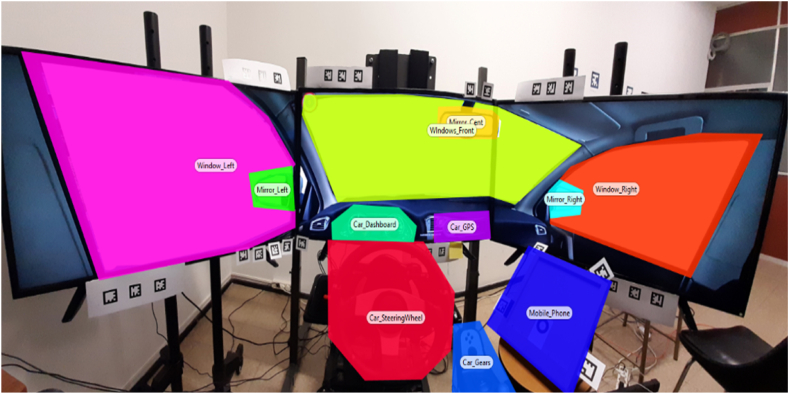
AOIs defined in the Tobii Pro Lab.

Moreover, the simulator has an internal timer that incorporates time information into the database and guarantees the reliability of recorded data regarding infraction times, and driver positions (inside or outside of each roundabout). Thus, the eye tracker captures the time of each Area of Interest (AOIs, Fig. 4) by displaying the eye tracker timestamp in real-time and facilitates the synchronization of both independent systems.

#### 2.1.3 Eye tracker

**Fig. 5:**
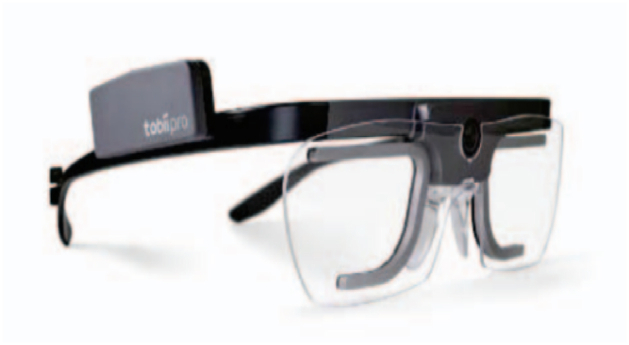
Tobii pro glasses 2.

To track participants’ eye movements in predefined sections of the roundabouts, a Tobii Pro Grasses 2 was used (Fig. 5). The latter consists of two cameras providing an accurate real-time data stream on the participants' gaze behavior at a sampling rate of 100 Hz. To improve the quality of the tracking system, many markers were added to the screen frame to visually connect the Tobii eye tracker to the real environment. This is because the virtual reality environment of the driving simulator is frame-changing and cannot be used as a reference for tracking.

### 2.2 Participants

In order to be selected to take part in the experiment, participants had to have a valid driving license, have no visual impairments, and be physically able to conduct the experiment.

A total of 43 participants signed an informed consent form approved by the Ethics Committee of the University of Burgos (Spain) and took part in this study. Among the 43 participants, there were 30 males and 13 females. The sample included young, middle-aged, and older drivers (ages ranged from 19 to 56 years old), with a mean and standard deviation of 23 and 6.85 years, respectively. In addition, the driving experience of the participants was determined by number of years of driver's license acquisition (ranging from 0 to 38 years). The driving frequency and number of Km the participants drove were also collected (Table 1).

**Table 1:** Summary of participants’ characteristics.

Variable	Category	Percentage (%)
Age (Years)	Y ≤ 20 20<Y ≤ 30 Y > 30	38.6 % 56.8 % 4.5 %
Gender	Male Female	72.7 % 27.3 %
Years with Valid Driving License (Years)	≤1 2 ≥3	25 % 29.5 % 45.5 %
Driving Frequency	Yearly Monthly Weekly Daily	2.2 % 13.3 % 42.2 % 42.2 %
Kilometers Driven (Km)	0–5000 5000–10,000 10,000–15,000 15,000–25,000 25,000–40,000 >40,000	48.9 % 24.4 % 17.8 % 8.9 % 0.0 % 0.0 %
Enjoyment of Driving	No A little bit Normal Yes Very much	0.0 % 8.9 % 11.1 % 46.7 % 33.3 %

### 2.3 Design and overview

#### 2.3.1 Procedure

Participation in this study was voluntary and participants were informed about the objectives of the study and told that they could quit the experiment at any time in case of any kind of discomfort. Experiments were conducted in several stages. In the first stage, participants were informed about the purpose of the study, briefed on various procedures, and given experimental instructions.

In the second stage, all participants completed a questionnaire regarding their demographics (age and gender), years with a valid driving license, driving frequency, and whether they like driving or not. Afterward, the participants took a practice drive for a few minutes to get familiar and comfortable with the virtual reality environment and different devices.

In the third stage, each participant completed two driving experiences. The first experience was a normal driving situation in which participants drove through a virtual urban road. The second experience was a distracted driving condition in which participants were preoccupied with mobile phone-related distractions.

#### 2.3.2 Experiment setup

**Fig. 6:**
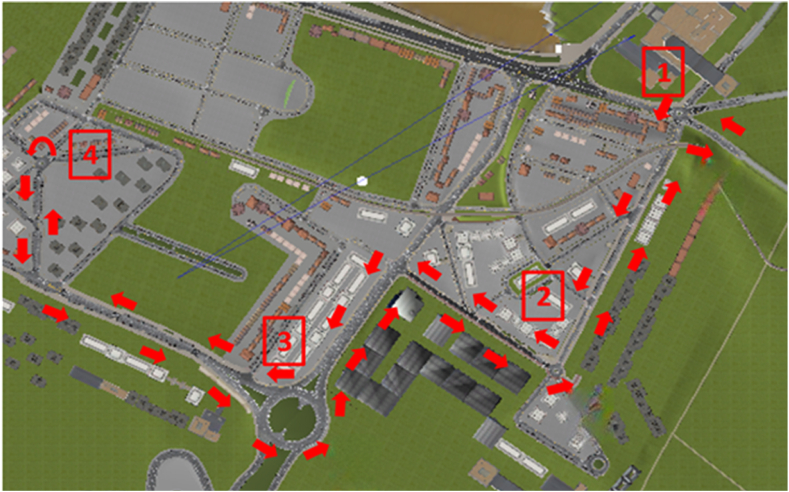
Aerial view of the virtual urban road.

All participants drove the same general scenario under the same driving conditions in the driving simulator, clear weather, daytime driving, and random but stable traffic flow. The driving scenario involved navigating a multi-lane urban road with four roundabouts (Fig. 6). Due to the road layout, three of the roundabouts were run twice. The course began at 1, went to 4 which was circled, and then back through the previous three roundabouts (3, 2, and 1).

**Fig. 7:**
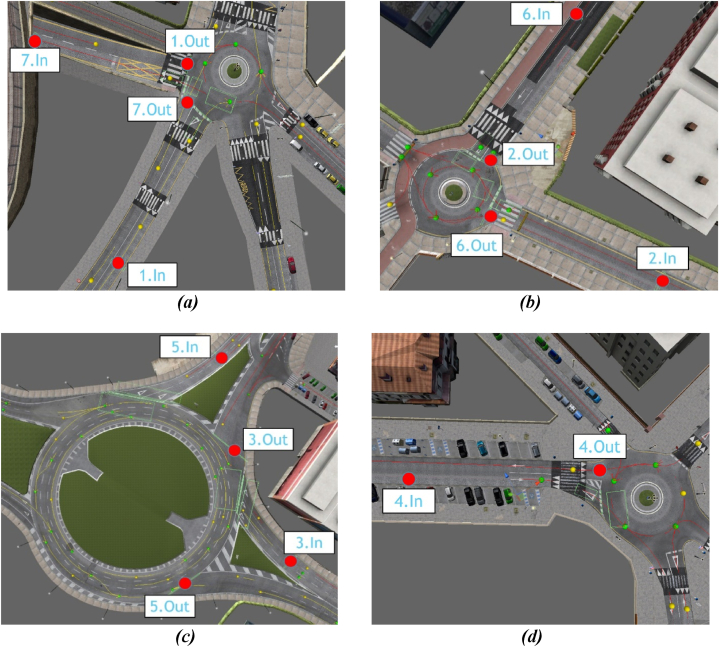
Detailed information about the entry and exit of each roundabout in the route where data was gathered: (a) roundabout (1); (b) roundabout (2); (c) roundabout (3); and (d) roundabout (4).

This study aimed to explore drivers’ behaviors at specific sections of roundabouts while experiencing distractions induced by mobile phone. The selected sections of roundabouts for the present experiment are shown in Fig. 7. Among the roundabouts examined, roundabouts (1), (2), and (4) are smaller in size, while the roundabout (3) is larger. Moreover, roundabout (3) features three lanes, whereas the others have only one lane.

**Fig. 8:**
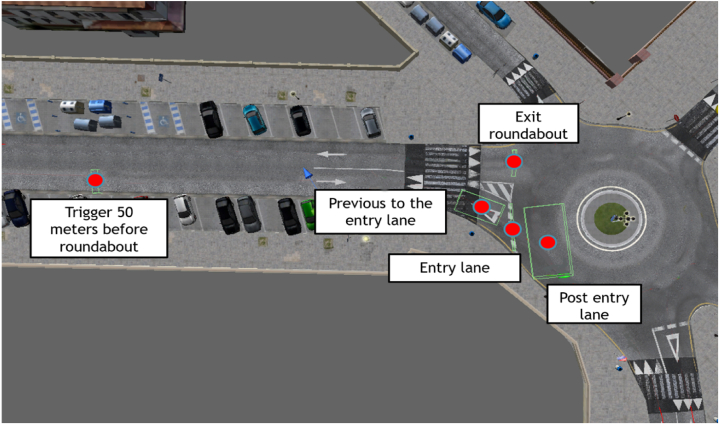
Spatial distribution of Locations of Interests (LOIs)'s of the third roundabout.

Previous research [20,27,32] showed that traffic crashes at roundabouts often result from drivers' failure to yield in entry lanes. To accurately capture the moments when drivers enter roundabouts, five triggers on Locations of Interest (LOIs) were integrated along each roundabout's entry and exit routes within the simulator (Fig. 8). These triggers on the LOIs serve to pinpoint the precise instant when a driver enters a roundabout. The first trigger was set approximately 50 m before the roundabout, providing an early indication. The second trigger was placed just before the entrance lane, alerting drivers to their imminent entry. The third trigger marked the beginning of the entrance lane, while the fourth trigger was positioned after the entrance lane to capture the transition onto the roundabout. Finally, the last trigger was placed at the exit line, marking the point of departure from the roundabout. The placement of these triggers was carefully chosen to provide comprehensive data on driver behavior at various critical points of roundabout navigation. The route of the experiment was designed with consideration of these LOIs.

### 2.4 Data collection procedure

#### 2.4.1 Experiment procedure

Before each driving session, an experimenter instructed the participants, mounted the eye tracker device onto their heads to track the eye movements and gaze patterns, and initiated the recording using the Tobii Glasses Controller software. At the end of each driving session, the experimenter stopped the recording and removed the eye tracker.

During the distracted driving session, the experimenter called the participants and exchanged conversation with them. Participants also had to respond to several WhatsApp messages and use Instagram. Similar questions and topics were discussed with all participants. These tasks were performed using a specific mobile phone provided for the experiment. The latter was placed in the simulator cockpit to the right of the steering wheel where it is usually mounted while driving.

#### 2.4.2 Study variables

Given the experiment design, the Tobii Pro Lab was configured with nine (09) AOIs, namely, left- and right-side windows, windshield, left- and right-side mirrors, rearview mirror, dashboard, steering wheel, and mobile phone.

In this study, from the data gathered, only changes in fixation on the left side mirror and window were considered. Fixation refers to the visual gaze focused on a particular location for a period of time while processing visual information [33,34]. In the particular case of this study, to homogenize the raw fixation data among the different types of roundabouts, the percentage of the total time spent in the roundabout, including all trigger points (shown in Fig. 8), was calculated.

The simulator records the telemetry data for each experiment into a SQLite database, allowing gathering a large amount of valuable data on the drivers’ driving performance. In fact, the simulator covers 28 different telemetry records related to the vehicle conditions at any given time, for instance, speed, control status, accelerations, etc. In this regard, there are up to 87 different conditions in which the simulator registers a penalty, i.e., exceeding speed limits, flashing lights, incorrect use of lights, crossing over continuous lines, going to the side of the road, etc. Infractions and driving violations of the drivers were extracted from the simulator records.

According to the study objectives, additional dependent variable sets were considered, namely: socio-demographic variables (age and gender), roundabout size, number of cars in the roundabout, and mobile-induced distractions.

### 2.5 Data analysis

In order to analyze the differences between the mean values, several alternatives to the standard Student's t-test [35] were considered to take into account the sample size (z-test, [36]), the applicability of the ANOVA Bartlett [37]; Arsham & Lovric [38] and the existence of two [39] or more (Kruskal-Wallis test, [40]) groups. In all cases, p-values were estimated using the functions included in the Statistics R package [41] to determine the statistical significance of the results obtained.

In this sense, Table 2 shows the results of comparisons among the variances of the different groups defined for the analysis proposed in this study to fulfill the hypothesis required for the application of the ANOVA analysis. In most cases, the null hypothesis can be rejected with the conclusion that, from a statistical point of view, the variances of each group considered are distinctly different. As a result, it wouldn't be appropriate to apply an ANOVA in this case and other tests should be used such as Wilcoxon and/or Kruskal-Wallis tests, depending on the number of groups considered. Based on these results the Kruskal-Wallis has been finally used to compare the means.

**Table 2:** Bartlett's test results (p-values) for different comparisons (i.e., with distractions vs. without distractions, large vs. small roundabouts, number of cars in roundabouts), and the different AOIs considered in this study. Cases in which the null hypothesis cannot be rejected are highlighted in bold.

	L. M.	L. W.	R. M.	R. W.	RV. M.	WS	W	D	M P
Distractions	7e-27	9.7e-7	7e-73	5e-103	7e-21	0.05	0.002	1.3e-10	**0.96**
Size	1.4e-4	**7.9e-1**	1.4e-11	**6.1e-1**	1e-19	1e-4	0.006	2.4e-4	9.1e-118
Cars	5e-24	1.4e-5	0.00	0.00	0.00	**0.30**	0.00	5e-21	0.00

**Note:** L.M: Left Mirror, L.W: Left Window, R.M: Right Mirror, R.W: Right Window, RV.M: Rear-View Mirror, WS: Windshield, W: Wheel, D: Dashboard, M.P: Mobile Phone.

Note that the probability of rejecting the null hypothesis as true is 0.05 considering the number of hypothetical tests used and the significance level considered (95 %). In that sense, it might be found that one of the 24 tests listed in Table 2, Table 3 would be rejected if the null hypothesis is true. For this reason, the compared means are also included in Table 3 to recognize this possibility.

**Table 3:** Kruskal-Wallis’ test results (H-statistic and p-value) for the two samples comparison of the largest roundabout (Fig. 7 panel c) and the remaining roundabouts considering the whole sample (first block), the laps without distractions (center block), and the laps with mobile use.

All	L. M.	L. W.	R. M.	R. W.	RV. M.	WS	W	D	M.P.
p-value	0.540	1.21e-01	**6.84e-11**	**6.87e-19**	**1.21e-09**	**3.7e-04**	**4.69e-07**	1.08e-01	**4.12e-04**
H	0.376	2.409	**4.26e+1**	**7.88e+1**	**3.70e+1**	**1.27e+1**	**2.54e+1**	2.590	**1.25e+1**
Mean L	4.346	9.668	1.05e-01	2.18e-01	1.111	74.286	0.740	4.953	2.839
Mean S	2.077	8.230	8.31e-01	4.279	1.511	68.660	0.869	4.088	4.835
No distractions	**L. M.**	**L. W.**	**R. M.**	**R. W.**	**C. M.**	**WS**	**W**	**D**	**M.P.**
p-value	0.954	6.39e-01	**4.05e-07**	**6.40e-12**	**6.36e-07**	0.181	**2.45e-04**	8.08e-01	9.57e-02
H	0.003	0.219	**2.57e+1**	**4.72e+1**	**2.48e+1**	1.787	**13.454**	0.059	2.777
Mean L	5.504	1.15e+1	0.000	9.29e-2	1.234	75.593	8.79e-01	5.378	4.56e-1
Mean S	2.245	8.437	0.598	4.434	2.040	74.451	8.14e-1	4.274	2.54e-1
Distractions	**L. M.**	**L. W.**	**R. M.**	**R. W.**	**C. M.**	**WS**	**W**	**D**	**M.P.**
p-value	0.425	0.076	**3.32e-5**	**1.54e-8**	**2.54e-4**	**3.58e-4**	**0.001**	**3.581e-2**	**2.164e-4**
H	0.636	3.144	**1.72e+1**	**3.20e+1**	**1.34e+1**	**1.27e+1**	**1.19e+1**	**4.406**	**1.37e+1**
Mean L	3.109	7.730	2.17e-1	3.51e-1	9.80e-1	72.889	0.590	4.498	5.388
Mean S	1.903	8.018	1.071	4.120	9.66e-1	62.679	0.927	3.896	9.565

**Note:** The mean values for the large roundabout (Mean L) and the small roundabouts (Mean S) were also included. The statistically significant p-values were highlighted in bold. The nine AOIs have been included in the table: Left Mirror (L.M.), Left Window (L.W.), Right Mirror (R.M.), Right Window (R.W.), Rear-View Mirror (RV.M.), Windshield (WS), Wheel (W), Dashboard (D), and Mobile phone (M.P). The degrees of freedom (DoF) corresponding to the Kruskal-Wallis’ test were not included since they are a constant parameter (DoF = 1) as the mains of the two groups were compared throughout the test.

Two types of figures were considered to present the results. On the one hand, the boxplot represents a box that gives the range between the 25th and the 75th percentiles for each value of the X-axis plot when the subsample is associated with the corresponding X-value while the Y-axis represents the fixation percentage. The horizontal line inside the box reflects the median value whilst the vertical lines outside of the box define the range established for the outliers. Points outside of these ranges are the subsample outliers. On the other hand, a heat map with the p-values is used to show the statistical significance of the differences between the means of the different parameters considered. In particular, the fixation on the left window and mirror is usually considered according to different parameters (i.e., number of penalizations, license years, etc.), leading to a symmetrical matrix. In the case of distractions, comparing the left window/mirror fixation means of two experiments with and without mobile phone use yields an asymmetric heat map. Note that 95 % statistical significance corresponds to the first color defined in the heat map.

Finally, the effect of the roundabout size on the average AOIs’ fixation was considered across samples, with and without mobile phone use. In particular, the largest roundabout (Fig. 7 panel ***c***) was compared with the remaining roundabouts.

## 3 Results and discussion

First, the applicability of the ANOVA for the study analysis was studied obtaining that the variance of the fixation on the 9 AOIs of each group defined for the different comparisons considered in this work was, for most of the cases, statistically different, reflecting a great heterogeneity of the sample among groups. As a result, the Kruskal-Wallis test was considered instead other more common options as the standard Student's t-test. Note that, since two groups were consistently compared, the degrees of freedom for this test remained consistently equal to one.

Second, changes in the AOIs fixation, based on driving conditions, were analyzed considering the number of cars and the size of roundabouts.

### 3.1 Fixation on left mirror and window and number of cars

**Fig. 9:**
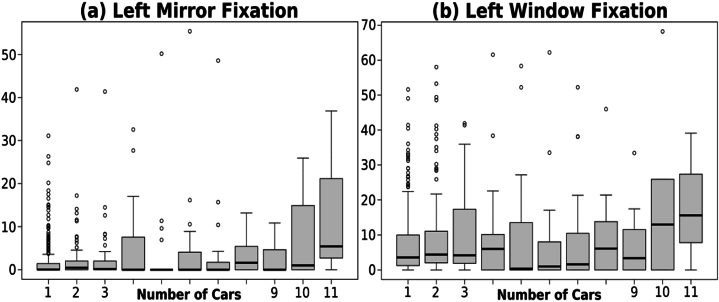
Fixation on the **(a)** left mirror and **(b)** window depending on the number of cars. Note that the title of each figure describes the specific fixation represented by the Y-axis.

The results indicated that fixation on the left mirror and window tended to escalate in tandem with the increasing number of cars inside the roundabout. However, it's is crucial to note that the statistical significance of obtained differences was somewhat constrained by the sample variability, as depicted in Fig. 9. Furthermore, upon closer examination of the fixation values, it became evident that drivers paid more attention to the left window than to the left mirror.

These findings could be explained by the fact that, while the left mirror primarily serves as a tool for monitoring adjacent lanes and blind spots, the left window offers a direct and a broader view of the road environment including incoming flow of traffic inside the roundabout. Therefore, drivers prioritized their visual attention towards this area to maintain situational awareness and anticipate any risks as it provided richer perceptual information.

### 3.2 Fixation on left mirror and window and roundabout size

Table 3 shows the results (H-statistic and p-value) of the Kruskal-Wallis’ test for the comparison of the AOIs’ fixation mean value of the largest roundabout (Fig. 7 panel c) and the smaller ones. Note that degrees of freedom associated with the test are constant and equal to 1 since all comparisons involve two groups. Therefore, this parameter was not included in Table 3. In order to better interpret the p-values, Table 3 shows the AOIs fixations for the largest roundabout and smaller ones for the three cases (all the sample and laps with and without distractions). Significant differences are obtained for the central and right parts of the car, with p-values less than 0.05 in the three cases for the right mirror, right window, rear-view mirror, and the wheel, which are more relevant for smaller roundabouts. Moreover, although not statistically significant, in all cases, the fixation on the left mirror is higher for the largest roundabout than for the smaller ones, whilst the differences in the left window fixation increase when there are no distractions in favor of the largest roundabout.

Right elements of the car garnered more visual attention in smaller roundabouts, in both baseline and distraction conditions. This could be explained by the fact that the drivers might adjust their scanning patterns based on the spatial constraints and traffic dynamics inherent to small roundabouts. Moreover, in smaller roundabouts, maneuvers were typically executed with less room for errors; therefore drivers allocated less attention to left window and left mirror. Furthermore, the fixation on the windshield and mobile phone was closely related to the use of a mobile phone as the differences obtained faded away when there were no distractions. This behavior could be expected as the drivers were switching their view between the mobile phone and the road, paying less attention to the rest of the AOIs.

These findings relate to Dong et al. [42] study comparing the effect of the mobile phone position on the drivers’ fixation and driving performance. The authors found that placing the mobile phone above the AC vent prompts drivers to switch their heads between the road ahead and the mobile phone. This behavior increases the glance range and prolongs fixation times.

Fixation on the left mirror was higher for the largest roundabout compared to smaller ones, particularly in the presence of mobile phone distractions, offers insights into how cognitive and visual load and environmental factors influenced the gaze behavior of the drivers. Indeed, induced distractions related to the mobile phone use reduced driver's awareness to incoming traffic flow inside the roundabout and blind spot and limited their ability to comprehend visual scanning information resulting in decreased fixation on critical areas (i.e., left mirror and window). Moreover, the heightened fixation to left window in larger roundabout, in the absence of mobile phone distraction, suggested that non-distracted drivers adopted a more cautions and a proactive approach to visual monitoring to cars inside the roundabout to ensure a safer navigation.

These findings relate to Rasanen & Summala [43] study exploring car drivers' adjustments to cyclists at roundabouts. The main findings confirmed that the drivers’ behaviors depend on the size of the central island. Interestingly, the authors found that the drivers approach the roundabouts, with large central islands, at a lower speed, which in fact allows them to adequately adjust their visual search pattern. Similarly, Vetturi et al. [44] reported that drivers slow down when traveling through large roundabouts and the level of attention is greater, i.e., up to 91 %.

### 3.3 Fixation on left mirror and window and number of infractions

The analysis of the number of infractions and gaze fixation reveals a significant relationship between the AOI and infractions. To ensure a sufficient sample size for each class, infractions committed at all roundabouts were grouped into dozens (1 mean value from 0 to 12, 1 from 13 to 24, and so on).

**Fig. 10:**
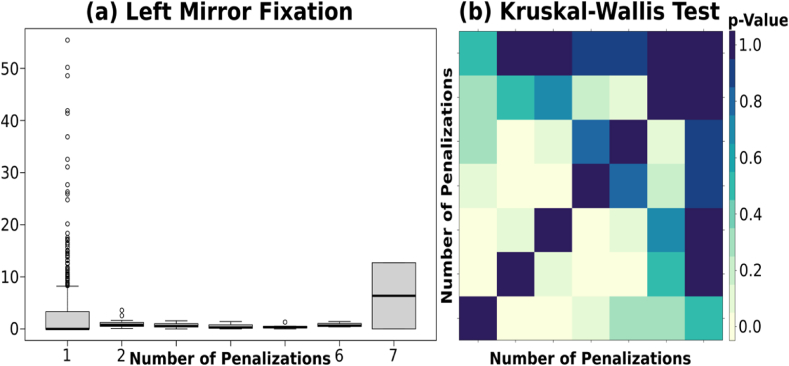
**(a)** Infractions related to the fixation on the left mirror **(b)** p-values for the Kruskal-Wallis test comparing the fixation on the left mirror at different numbers of infractions.

Results in Fig. 10 showed the statistical significance that the more participants fixed their gaze into the left mirror while driving in roundabouts, the fewer infractions they committed. Note that the case with more penalizations has only two valid observations, 0.00 and 12.71, leading to the anomalous behavior observed. The left mirror serves as a key tool for monitoring adjacent lanes and blind spots, enabling drivers to make informed decisions and execute maneuvers safely in roundabouts. This is coherent with the literature related to drivers’ behaviors when traveling through roundabouts. A previous study conducted by Verma et al. [45] showed that dual-task driving leads to poor driving performance in terms of poor lane keeping. Similar conclusions were drawn from a simulator study of the effects of mobile phone use on the driving performance of young drivers [21]. This study revealed significant differences in vehicle control (i.e., lateral distance and hard shoulder line violations) between baseline and mobile phone distraction conditions.

Moreover, drivers who allocated more visual attention to the left mirror were likely to demonstrate greater attention to roundabout features and heightened vigilance regarding surrounding traffic. Thus they were less likely to commit infractions, such as failing to yield or give way [46].

### 3.4 Fixation on left mirror and window and driving experience

**Fig. 11:**
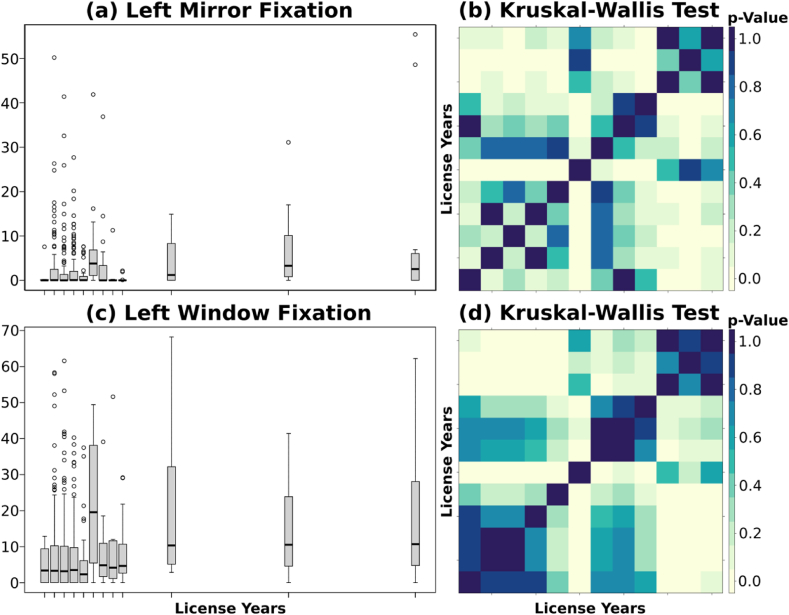
***(a)*** License years associated with left mirror fixation ***(b)*** p-values for the Kruskal-Wallis test comparing the left mirror fixation by license years ***(c)*** license years associated with left window fixation ***(d)*** p-values for the Kruskal-Wallis test comparing the left window fixation by license years.

The study results uncovered an important finding pertaining to the driving experience of the participants. Remarkably, the older a driver's license is, the more often participants demonstrated a tendency to look more frequently at both the left window and mirror. This trend was consistently evident and statistically significant across numerous cases as depicted in Fig. 11. Moreover, despite the variability within the sample which affects the statistical significance of the results, similar conclusions were obtained when considering other variables related to the drivers' experience, including frequency of driving, distance driven, and love for driving.

The heightened situational awareness and anticipation skills of experience drivers resulted from the adaptive nature of visual scanning strategies of experienced drivers gained with more years of driving experience, Moreover, with time and practice, drivers often refine their driving habits and adopt more efficient gaze behavior. Furthermore, experienced drivers had the ability to instinctively prioritize their visual attention towards left mirror and window, the most relevant areas, to verify neighboring vehicles and traffic flow in the roundabout and pay less attention to other in-vehicle elements and distractions. Consequently, they maintained a high level of awareness and responsiveness to navigate roundabouts safely. These findings are consistent with Konstantopoulos et al. [47] study that tracked gaze behavior and driving performance of driving instructors. The outcome of this latter showed that experienced drivers have a longer fixation period on side mirrors, shorter processing time, better sampling rate, and wider scanning of the environment. In line with these conclusions, Falkmer & Gregersen [48] noted that inexperienced drivers focus their attention on in-vehicle objects and limited areas inside and outside the vehicle.

### 3.5 Gaze behavior of the drivers under baseline and distraction conditions

**Fig. 12:**
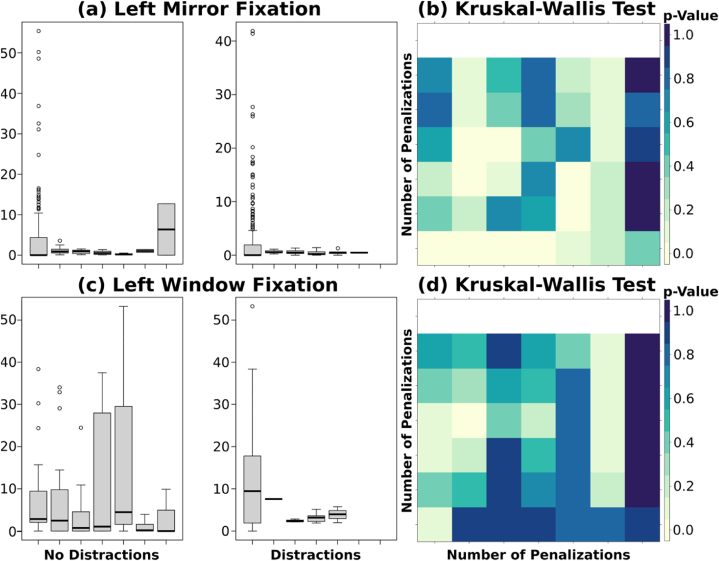
**(a)** Comparison of infractions associated with left mirror fixation with respect to the baseline and distractions' conditions **(b)** p-value from the Kruskal-Wallis test comparing the fixation on the left mirror under baseline and distractions' conditions with respect to the different number of infractions **(c)** Comparison of infractions under baseline and distractions conditions with respect to the left window fixation **(d)** p-value from the Kruskal-Wallis test comparing the left window fixation under baseline and distractions conditions with respect to the different number of infractions.

In the context of analyzing gaze behavior under baseline and distraction conditions, the findings underscored the relevance of drivers' visual attention in the absence of mobile phone distractions. Moreover, the analysis revealed that drivers exhibited a statistically significant higher frequency of glances towards their left mirror, although there was greater uncertainty for the left window. However, when examining left window fixation during the distraction's lap, the main conclusion drawn from Fig. 12 is that a decrease in infractions coincides with an increase in left window fixation. For this analysis, the same seven groups as those used in previous penalizations analysis were used.

Mobile phone use behind the wheel diverted drivers’ attention away from the driving task, and adversely impacted their gaze behavior particularly their focus on the left window and mirror when navigating roundabouts. This potentially increased the risk of errors, infractions, or lapses in judgment. Indeed, in the baseline condition, the drivers maintained vigilance towards the left mirror which allowed them to gather essential information about the road environment, including the presence of other vehicles or pedestrians. This heightened situational awareness to left mirror and window facilitated early detection of potential risks, and aided proactive hazard avoidance strategies, thereby reducing the likelihood of committing infractions.

These findings relate to a previous study by Haque et al. [20] compared drivers’ behavior with and without mobile phone-induced distractions, when traveling through roundabouts. The authors reported that distracted drivers accepted smaller safety margins than non-distracted drivers. Likewise, Azimian et al. [32] compared the total fixation of drivers at roundabouts, under baseline and mobile phone distractions, and concluded that mobile phone use reduced fixation duration in all rearview mirror, windshield, left-side mirror, and window and passenger-side mirror and window.

### 3.6 Study limitations and directions for future research

In summary, this study was designed to safely and efficiently collect a very large amount of data on gaze behavior and driving performance of drivers at roundabouts under baseline and mobile phone distractions conditions. However, there are potential limitations due to the nature of the present study which used a driving simulator that cannot capture all possible drivers' behaviors under distraction in a real-world driving environment. Moreover, the present study selected only four, one and three-lanes, roundabouts. The design of driving simulator parameters plays an important role in research results. In fact, the nature, type, and specifications of roundabouts directly affect the stimuli presented to the drivers and, consequently, the level of realism. Further research should look into drivers' performance at multilane and spiral roundabouts and consider different simulator configurations of traffic and road users to gain further insight into the severity of mobile phone use while entering, driving in, and exiting a roundabout. In addition, as the experience gained by the driver on the first lap may influence their behavior on the second lap, future studies should consider a random selection of the lap order (with and without mobile phone distractions) to avoid bias and obtain more robust conclusions. Furthermore, this study's design was limited to only analyzing the drivers' experience. In fact, driving behavior and driving style depend on the driver's particularities, for instance, risk perception, gender, age, personality traits, emotional and behavioral conditions, attention, eye-gaze dynamics, body movement, and gestures [49,50]. Future studies should broaden the context of the current paper and examine other factors that may affect the drivers' performance. Particular attention could be paid to differences in cognitive control between young and older drivers and impacts on their driving performance.

Finally, given the fact that roundabouts are designed to congregate different road users, steering safely through roundabouts requires drivers to consider many aspects other than checking their left side mirror and window. Thus, future research may look into other behaviors such as the use of turn signals, yielding rate, gap acceptance, and braking behavior, and may explore the influence of other distraction conditions (texting, operating a music player or radio, and eating while driving).

## 4 Conclusions

In light of the growing evidence available on driving distraction being a cause behind traffic crashes, the present paper attempted to provide in-depth insight into mobile phone-related driving challenges at roundabouts and the potential infractions of the drivers. By tracking drivers' eye movements and examining their driving performance under mobile phone distractions, the present study provided new insight and perspectives for behavioral and road safety research. Notably, geometric designs, i.e., roundabout size, affect drivers' fixation on the left mirror and window. Moreover, under distraction conditions, the drivers' fixation on the windshield and mobile phone increases significantly compared to the rest of AOIs. Interestingly, the results showed that experienced drivers paid more attention to their left while driving through roundabouts, and that more fixations on the left mirror and window led to fewer infractions. It's worth noting that, although the order of laps can lead to underestimating the differences between the two laps, implementing random selection could enhance statistical significance. Additionally, different driving conditions ensure the relative independence of each lap, thereby mitigating the influence of experience gained during the initial lap.

Drawing from these findings, a range of both active and passive safety measures can be added. For instance, installing signals on approach roads to roundabouts to remind the drivers to check for incoming cars on their left before entering. More active measures could involve integrating alerts into GPS navigation software, allowing for customized messages tailored to roundabout type, driver age, and their seniority.

## Institutional review board statement

The study was conducted according to the guidelines of the Declaration of Helsinki, and approved by the Ethics Committee of Burgos University (protocol code IR 17/2020 of May 28, 2018).

## Funding

This project was funded by 10.13039/501100002924FEDER European Regional Development Fund (Fondo Europeo de Desarrollo Regional—Junta de Castilla y León, Spain), grant number BU300P18. Title: Modelización mediante técnicas de "machine learning" de la influencia de las distracciones del conductor en la seguridad vial. Diseño de un sistema integrado: simulador de conducción, "eye tracker" y dispositivo de distracción.

## Data availability statement

Data will be made available on request.

## CRediT authorship contribution statement

**Wafa Boulagouas:** Writing – original draft, Investigation, Conceptualization. **Ortega Carlos Alberto Catalina:** Investigation, Writing – original draft. **Miguel Angel Mariscal:** Writing – review & editing, Supervision, Project administration. **Sixto Herrera:** Writing – review & editing, Validation, Supervision, Software, Methodology, Formal analysis, Data curation, Conceptualization, Investigation. **Susana García-Herrero:** Writing – review & editing, Writing – original draft, Validation, Supervision, Resources, Project administration, Investigation, Funding acquisition, Formal analysis, Conceptualization.

## Declaration of competing interest

The authors declare that they have no known competing financial interests or personal relationships that could have appeared to influence the work reported in this paper.
