# Supplementary material for: Effects of mobile phone-related distraction on driving performance at roundabouts: Eye movements tracking perspective
Source: Heliyon. 2024 Apr 10;10(8):e29456. doi: 10.1016/j.heliyon.2024.e29456 (PMC11040046; doi:10.1016/j.heliyon.2024.e29456)
Supplement: Multimedia component 1 [file mmc1.docx]

**Table.** Summary of previous related studies

| **Driving performance parameter** | **Location** | **Type of the road** | **Participants** | **Distraction** | **Type of the Study** | **Main findings** | **Reference** |
| --- | --- | --- | --- | --- | --- | --- | --- |
| Use of turn signals | Doha city | Two-lane roundabouts | 400 and 415 | / | Observation | 23% of incoming vehicles and 18% of outgoing vehicles used turn signals | (Muley et al., 2022) |
| Yielding rate and gaze behavior | / | Multilane roundabouts | 45 | / | Driving Simulator + Eye Tracker | Implementation of beacons increased the drivers’ attention to beacons and pedestrians along the road. | (Salamati et al., 2012) |
| Gaze behavior (Total fixation duration) | / | Multilane roundabouts | 45 | Mobile phone | Driving Simulator + Eye Tracker | Careless driving due to mobile phone use.  Reduced fixation duration in all of the considered areas of interest (front mirror, windshield, driver-side mirror, driver-side window, passenger-side mirror, and passenger-side window). | (Azimian et al., 2021) |
| Use of signals and give the way | Riyadh | Roundabouts | -750 drivers observed  - 384 participants | / | Observation + Questionnaire | -Most reported unsafe behaviors are entering the roundabout without giving way and exiting it without flashing.  -There is insufficient knowledge of driving rules at roundabouts. | (Al-Saleh & Bendak, 2012) |
| Eye movements and gaze concentration | / | Two-lane roundabouts | 24 participants | / | Real driving test + 3 eye trackers, GPS, CAN bus sensors. | -Drivers spent 28.36% of their time looking at non-driving areas.  -A significant number of drivers entered a roundabout without checking the lanes. | (Abbasi et al., 2022) |
| Eye movements, speed | Italy | Roundabout | 10 participants | / | Real driving test + ASL Mobile Eye-XG device | -Implementation of zebra crossing markings and “Yield here to pedestrians” vertical signs and the introduction of media refuge islands increased visual attention of motorists to pedestrians (pedestrian crossings were perceived from a longer distance). | (Vignali et al., 2020) |
| Eye movements | / | Urban routes. | 21 participants | / | Simulator + Eye Tracker | -Driving instructors (experienced drivers) had a higher sampling rate, shorter processing time, and a wider scanning of the road.  -Experienced drivers got a longer fixation on side mirrors than learner drivers. | (Konstantopoulos et al., 2010) |
| Eye movements, response time, speed at the yellow light, stopped or ran the yellow light. | / | Intersection | 24 participants | / | Simulator + Head Mounted Optics Module + Magnetic Head Tracking Hardware. | - Use of in-vehicle signs increased stopping rates for both young and older drivers.  -Younger drivers were found to look at the in-vehicle signs for longer durations than older drivers.  -Older drivers were found to slow down and stop correctly when approaching the intersection compared to younger drivers. | (Caird et al., 2008) |
| Eye movements (eye position), unsafe behavior | / | 16 scenarios including intersections | 72 participants | / | Driving Simulator+ Eye Tracker | - The way novice drivers scan made them fail at acquiring information about potential risks.  -How novice drivers scan negatively affects the way they deal with risks.  -There were significant age-related differences in risk perception. | (Pradhan et al., 2005) |
| Eye movements (Total fixation duration, fixation transition probability), driving behavior at stop-sign-controlled grade crossings | / | Grade Crossings | 41 participants | / | Driving simulator+ Eye tracker | -Improved design of signs and audio warning made the grade crossing safer.  -Improvements enhanced stop compliance rate.  -Females were found to have a longer fixation duration on the STOP sign and a higher compliance rate than males.  -Females were found to have an earlier perception of the train approaching. | (Liu et al., 2022) |
| Gap acceptance performance, intersection driving performance, and traffic violation behaviors. | / | Rural thru-stop controlled Intersections | 105 participants | / | Driving simulator | - Original rural intersection-controlled warning signs (RICWS) proved to be safer than control intersections.  - Risk of stop sign violations increased among all the drivers in the case of the original RICWS.  -Original RICWS contributed to improving drivers’ gap acceptance performance. | (Tian et al., 2021) |
| Driving performance at the onset of yellow traffic lights | / | Signalized intersections | 78 participants | / | Driving simulator | -With the advanced information aids, the drivers tended to not cross intersections at the onset yellow light.  -With advanced information aids implemented, females were found more likely to run through the intersections at the onset of yellow light.  -With the advanced information aids implemented, the drivers made safer decisions at the onset of yellow light. | (Ali et al., 2021) |
| Perception of roundabouts safety and behaviors of users on the roundabouts | Poland | Mini, single-line, multi-lane, semi-two-lane, turbo roundabouts, and roundabouts with the untypical shape. | 1800 respondents | / | Survey | - 87% of the respondents reported that they pay attention to vertical and horizontal markings while driving.  -More than 50% of the drivers confirmed that they yield the right of way to pedestrians at entries and exits of roundabouts.  - 73% of the respondents said that the design of the central island influenced the behaviors of the drivers and 72% agreed that the central island defines the speed of moving through roundabouts. | (Macioszek & Kurek, 2020) |
| Gap acceptance | / | Roundabouts | 32 participants | Mobile phone | Driving simulator | - Reduced reaction distance in distracting conditions compared to the baseline.  - Under distractions, the initial speeds decreased and the drivers slowed down even more.  - Distracted drivers had a slower response time when accepting gaps. | (Haque et al., 2016) |
| Speed, drivers’ behaviors towards traffic rules, effects of lane division on drivers’ behaviors | Poland | Turbo-roundabouts | / | / | Monitoring system (Footage videos) | -Drivers often illegally change lane when exiting a roundabout.  -Implementation of lane separations could slow down drivers entering roundabouts.  -Higher speed is registered in the inner lane.  -Drivers in neighboring lanes with dividers registered slower speed.  -Without physical separations, drivers tended to speed up and drive over the lane edge. | (Chodur & Bąk, 2016) |
