# Supplementary material for: Effects of mobile phone-related distraction on driving performance at roundabouts: Eye movements tracking perspective
Source: Heliyon. 2024 Apr 10;10(8):e29456. doi: 10.1016/j.heliyon.2024.e29456 (PMC11040046; doi:10.1016/j.heliyon.2024.e29456)
Supplement: Multimedia component 2 [file mmc2.docx]

| **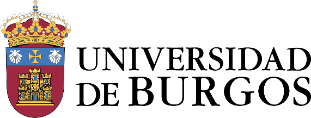***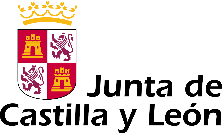* | *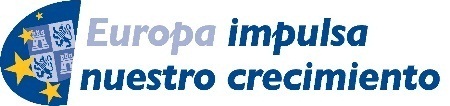* | *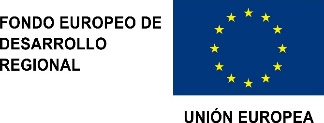* |  |
| --- | --- | --- | --- |

**Questionnaire**

| *Participant Information* |
| --- |
| 1. Conductor ID:   …………………………………………………………………………………   1. What is your age?   …………………………………………………………………………………   1. What is your gender?   □ Male □ Female   1. Number of years with a valid driving license :   …………………………………………………………………………………   1. What is the frequency at which you drive a car?   □ Yearly  □ Monthly  □ Weekly  □ Daily   1. What is the number of Kilometers you drive per year?   □ 0 – 5000  □ 5000 – 10,000  □ 10,000 – 15,000  □ 15,000 – 25,000  □ 25,000 – 40,000  □ > 40,000   1. Do you enjoy driving?   □ No  □ A little bit  □ Normal  □ Yes  □ Very much |
